# Co-occurrence of antibiotic resistance and virulence Genes in Methicillin Resistant *Staphylococcus aureus* (MRSA) Isolates from Pakistan

**DOI:** 10.4314/ahs.v22i1.57

**Published:** 2022-03

**Authors:** Ufaq Tasneem, Mahnoor Majid, Khalid Mehmood, Redaina, Fazal Ur Rehman, Saadia Andleeb, Muhsin Jamal

**Affiliations:** 1 Atta-Ur-Rahman School of Applied Biosciences (ASAB), National University of Sciences and Technology (NUST), Islamabad, Pakistan; 2 Department of Pharmaceutics, College of Pharmacy, University of Hail, Hail, Saudi Arabia Saudi Arabia; 3 Department of Pharmacy, Abbottabad University of Science and Technology, Havelian, Pakistan; 4 Department of Microbiology, Abdul Wali Khan University, Mardan, Pakistan; 5 Department of Microbiology, Faculty of Life Sciences. University of Balochistan, Quetta, Pakistan

**Keywords:** Methicillin resistant *Staphylococcus aureus*, Antibiotic resistance, Virulence, mecA, Pakistan

## Abstract

**Introduction:**

Methicillin resistant *Staphylococcus aureus* (MRSA) is one of the major human pathogen that is associated with hospital as well as community acquired infections and is responsible for huge amount of life-threatening diseases.

**Objective:**

Objective of the study was to determine MRSA prevalence, their antibiotic sensitivity patterns, frequency of virulence genes (sea, seb, sed, tst, hla, hld) and their co-occurrence with resistance marker mecA among Rawalpindi and its nearby regions of Pakistani clinical isolates.

**Methodology:**

The present study was carried out to identify the virulence and antibiotic resistance genes that co-occur in MRSA through polymerase chain reaction. Antibiotic sensitivity, presence of virulence genes and their co-occurrence with resistance marker mecA were analyzed.

**Results:**

These isolates were found resistant to number of antibiotics i.e. Amoxicillin (16.1%), Cefixime (48.38%), Doxycycline (27.415), Trimethoprim/sulfamethoxazole (37.09%), Clindamycin (30.64%), Erythromycin (83.87%), Penicillin (100%), Vancomycin (4.83%), Ciprofloxacin (70.96%), Tetracycline (20%), Linezolid (3.22%) and Fusidic acid (11.295). The frequency of antibiotic resistant gene (mecA) was 69.35% and that of virulence genes hla, hld, sea, seb, sed and tst was 100, 100, 53.2, 30.6, 3.2 and 24.2% respectively. Amongst all examined genes, hla and hld genes had the highest and sed gene had the lowest frequency. The maximum coexistence of genes was observed for hla+hld+mecA gene combination (42 out of 62 isolates).

**Conclusion:**

This study reports the presence of multidrug resistant, vancomycin-resistant and mecA negative MRSA isolates in infected patients of Rawalpindi and nearby regions of Pakistan that may have attributed to treatment failures, adaptability of new virulence characteristics and spread of antibiotic resistance.

## Introduction

*Staphylococcus aureus* is a Gram positive bacterium associated with skin and soft tissue infections (SSTIs), septic arthritis, abscesses, necrotizing fasciitis and food poisoning. The indiscriminate and irrational use of antibiotics resulted in emergence of resistance in clinically important pathogens culminating in limited treatment options, prolonged therapy, increasing costs and threatened success of surgical procedures. This increased resistance has struck at the very root of already poor healthcare system in developing countries, where burden of infectious diseases is much higher and patients either have limited access to medical facilities or treatment costs is high. Low socio-economic status, lack of personal hygiene, poor quality of drinking water and feeble general health attributes are leading to more severe outcomes for inhabitants of developing world^1^.

MRSA infections are emerging rapidly in hospitals and community consequently increasing MRSA-associated morbidity and mortality^2^. The common community-acquired MRSA infections are related to skin that may lead to necrotizing fasciitis, necrotizing pneumonia, infective endocarditis, and bone and joint infections^3^. The first strains of Methicillin-resistant *S. aureus* were reported during early sixties in England^4^. MRSA attains resistance to methicillin and similar antibiotics through acquisition of mecA gene that codes for penicillin-binding protein PBP2a. This gene is a part of 21–60 kb Staphylococcal cassette chromosome (SCCmec); a mobile genetic element^5^.

The development of resistance was mainly associated with transfer of resistance imparting genes from environmental and commensal organisms^6^. The relationship between antibiotics use and emergence of resistance has been established and attributed to two factors; the antibiotic sparing resistant population to thrive, and transfer of genetic component responsible for imparting resistance^7^. Intrinsic resistance serves as a mechanism to resist the effect of self-produced antibiotics or have possible use in central metabolic processes. However, selective pressure driven by antibiotic use has been the driving source in shaping the resistance mechanisms^7^, ^8^.

*S. aureus* pathogenic determinants are responsible for colonization, tissue damage and consequent pathologies^9^,^10^. *S. aureus* strains produce exotoxins like toxic shock syndrome toxin-1 (TSST-1), enterotoxins like sea, seb, secn, sed, see, seg, seh, and sei, exfoliative toxins like eta and etb and enzymes like nucleases, proteases, lipases, hyaluronidase and collagenase. All of these factors contribute in bacterial colonization^10^, subsequent inflammation and virulence^11^, ^12^.

The knowledge about prevalence of MRSA and SSTIs burden in a community can help devise effective intervention strategies for MRSA control including screening programmes, hygienic standards, proper disposal of hospital wastes, judicious use of antibiotics, sanitizing infective surfaces and public health awareness. Such data are scarce in developing countries like Pakistan so we intended to report MRSA prevalence among Pakistani clinical isolates, their antibiotic sensitivity patterns, frequency of virulence genes (sea, seb, sed, tst, hla, hld) and their co-occurrence with resistance marker mecA.

## Methodology

### Sample Collection

Among 62 samples including pus, sputum and urine, highest proportion of Methicillin resistant Staphylococcus aureus (MRSA) was collected from pus i.e. 59 while the lowest proportion was found in urine with only 1. Two MRSA strains were isolated from sputum of patients belonging to various age group and gender from Armed Forces Institute of Pathology, Rawalpindi, Pakistan. Informed consent of the subjects were taken and ethical approval was obtained from Institutional Review Board, Atta-ur-Rahman School of Applied Biosciences, National University of Science and Technology, Islamabad Pakistan. These clinical isolates were carried in vials (CORNING, 1.8 ml) to the laboratory in an ice box and stored in refrigerator at 4°C till further analysis.

### Sample Processing

The collected samples were processed within 24 hrs of collection using standard microbiology techniques. The strains were inoculated in LB media tubes and also spread on nutrient agar plates and incubated overnight at 37°C in aerobic atmosphere. The LB media tubes and plates were visualized very next day. All plates were examined for *S. aureus* by colony morphology. Glycerol stocks of samples were prepared using 800 µL of glycerol (99%) and 200 µL of 24 hrs incubated LB media containing dense bacterial growth, and stored at 20 °C.

### Antimicrobial susceptibility testing

Clinical and Laboratory Standard Institute (CLSI) guidelines were followed for media preparations, media selection, antibiotic discs' placement and measurement of zones of inhibitions. Bacterial growth from a pure culture plate was spread on fresh Muller Hinton agar plate. Antibiotic discs of known concentration (Table S1) were applied using sterilized forceps. Plates were incubated at 37°C in aerobic atmosphere. The zones of inhibition were interpreted using CLSI guidelines^13^.

Calculation of Multiple Antibiotic Resistances (MAR) Index MAR index was determined^16^. A MAR index for an isolate is calculated as:

$$\begin{array}{l} \text{MAR index was determined16. A MAR index for an isolate is calculated as:}\\ \begin{array}{cc} \text{MAR index =} & \frac{\text{Number of antibiotics to which isolate is resistant (a)}}{\text{Total number of antibiotics against which isolate was tested (b).}} \end{array} \end{array}$$

MAR value greater than 0.2 indicates high exposure to antibiotics. Bacteria having MAR Index > 0.2 originate from an environment where several antibiotics are used^17^.

### In-silico identification of virulence and antibiotic resistance genes

S. aureus genes coding for virulence factors were identified using Victor (http://www.phidias.us/victor) and VFDB (http://www.mgc.ac.cn/VFs/main.htm) databases. The genes for antibiotic resistant were identified by using ARDB (https://ardb.cbcb.umd.edu/) and CARD (https://card.mcmaster.ca/) databases. Primers for both the virulence and antibiotic resistance genes were obtained as reported previously^14^, ^15^ (Table S 2). These primer sequence specificities with required gene was determined using BLAST software.

### DNA extraction and PCR amplification

Bacterial DNA was extracted from overnight streaked plate. The growth was picked with sterile pipette tip and suspended in 100 µL of nuclease free water in eppendorf and mixed thoroughly to form a turbid solution. The tube was then given a heat shock of 40 min at 120 °C and was spun for 3–4 min until pellet was formed. The supernatant containing bacterial chromosomal and plasmid DNA was then collected in a sterile eppendorf and pellet was discarded. The template DNA presence in the supernatant was confirmed by loading on 0.8% ethidium bromide stained gel. The samples were placed in freezer until use. Table S3 gives the details of PCR master mix and Table S4 details the PCR conditions employed for both virulence and resistance genes profiling. The reaction mixture was thoroughly mixed using micro centrifuge (Edison, New Jersey and USA) and PCR amplifications were performed using SwiftTM MaxPro thermal cycler (Applied Biosystem, Foster city, USA).

### Agarose gel electrophoresis

To analyze the PCR products, 0.8% agarose gel was prepared in 1X TBE (10.8g Tris, 5.5g Boric acid, 4 mL of 0.5 M of Na2EDTA (pH 8.0) in 1 L) and was run in the same buffer composition. Around 2 µL of 6X loading dye was mixed with PCR product and 7 µL sample was run on the gel. Power supply (Wealtec, Sparks, USA) was set at constant current of 70 mA for 40 min. The gel was then visualized under UV transilluminator (Biometra, Gottingen, Germany) to see the amplified product. The PCR products were stored at -20 °C for further use. The genes were sequenced and checked for their homology with other reported genes using BLAST software (https://blast.ncbi.nlm.nih.gov).

## Results

Frequency of isolates on the basis of infection source Characteristic yellow to cream or seldom white coloured, 1–2 mm in diameter, slightly raised colonies mostly appearing in groups were identified as S. aureus. Among 62 samples, the highest number of MRSA isolates (n=59) were collected from pus, whereas just one isolate was obtained from urine and two from sputum.

### Frequency of MRSA isolates on the basis of age and gender

**Figure 1:**
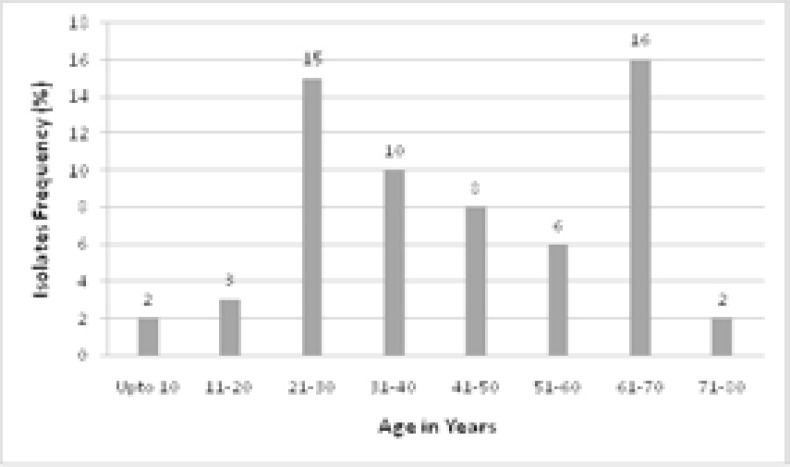
Frequency of MRSA isolated from patients of various age groups.

MRSA strains were isolated from patients of all age groups. Results were assessed on the basis of age division of 10 years. Highest frequency of MRSA associated infections were found in patients belonging to age group of 61–70 followed by age group of 21–30 years. Lowest frequency of MRSA infections was found in children less than 10 years of age (Figure 1). Male patients (75.9%) were more infected with MRSA as compared to the females (24.1%).

### Antibiotic sensitivity test

**Figure 2:**
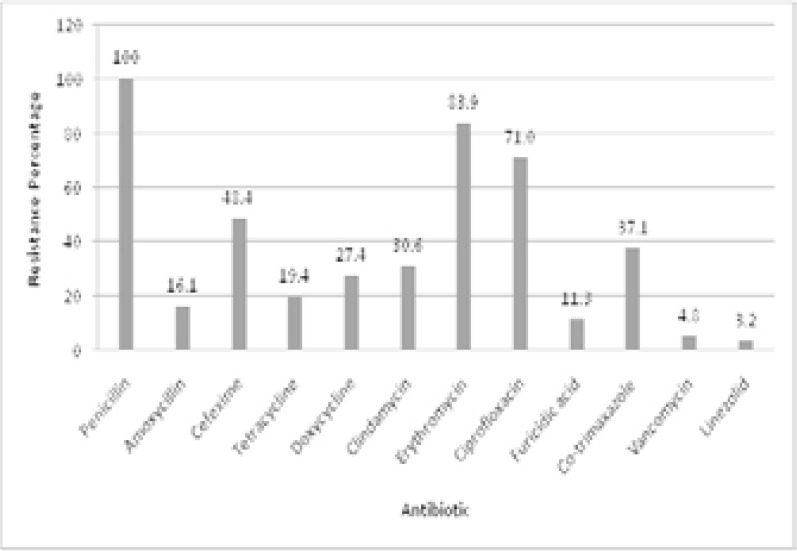
Percentage of resistant isolates against corresponding antibiotics.

All of 62 isolates were subjected to antibiotic sensitivity testing against 12 antibiotics using disc diffusion method. The highest resistance was found against penicillin and lowest against linezolid. Among the antibiotics from penicillins and cephalosporins group, penicillin showed the greatest resistance as all of 62 isolates were found resistant as compared to 16.1% (10 out of 62) resistance against amoxicillin with two isolates showing intermediate resistance. Thirty out of 62 (48.4%) MRSA isolates showed resistance against cefexime while remaining 32 were sensitive. Erythromycin followed the penicillin and showed higher resistance of 83.9% (52/62) followed by ciprofloxacin (71%, 44/62) and co-trimaxazole (37%, 23/62). Tetracycline was also among most sensitive antibiotics with only 19.4% (12/62) isolates showing resistance against it whereas the other member of the same class doxycycline showed 27.4% resistance (17/62). Clindamycin was also comparable to doxycycline with 19 isolates (30.6%) showing resistance against it. Least resistance was found against linezolid as only 2 isolates (3.2%) were resistant followed by 3 against vancomycin (4.8%) and 7 isolates resistant to furisidic acid (11.3%) (Figure 2).

### Calculation of Multiple Antibiotic Resistances (MAR) Index

**Figure 3:**
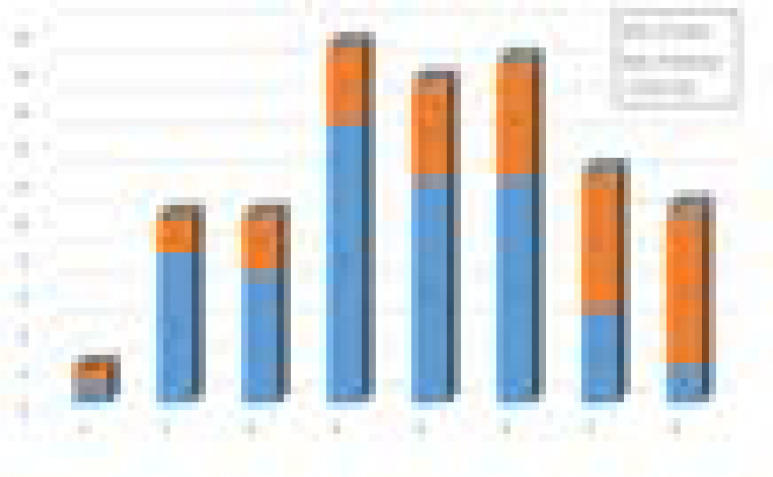
MAR index of MRSA isolates.

MAR of the bacterial isolates was determined. The MAR Index analysis (Figure 3) revealed that 46 isolates had very high MAR index value (>0.2). Seven isolates had MAR index of 0.2 while only 9 isolates had MAR values <0.2. Mean MAR value was calculated as 0.3619 which was considered as high.

### Molecular detection of resistance and virulence markers

The resistance marker of MRSA mecA was amplified using PCR, and successful amplicon was observed at 448bp with 100bp DNA marker. The overall prevalence was found to be 69.3 % while the remaining 31% lack this gene. The successful amplicons for virulence markers hla, hld, sea, seb, sed and tst genes were observed as 209 bp, 111 bp, 102 bp, 164bp, 278 bp and 326 bp, respectively using the 50bp DNA ladder as reference.

### Percentage occurrence of virulence and antibiotic resistance genes

**Figure 4:**
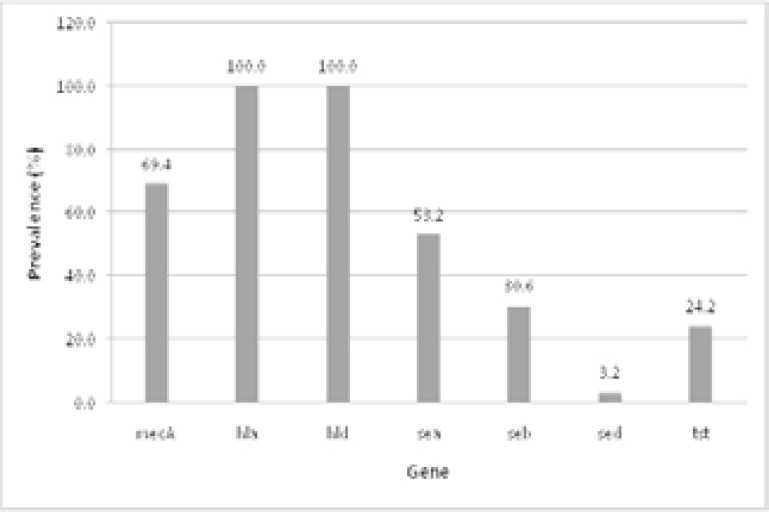
Percentage occurrence of virulence and antibiotic resistant genes in MRSA.

**Figure 5:**
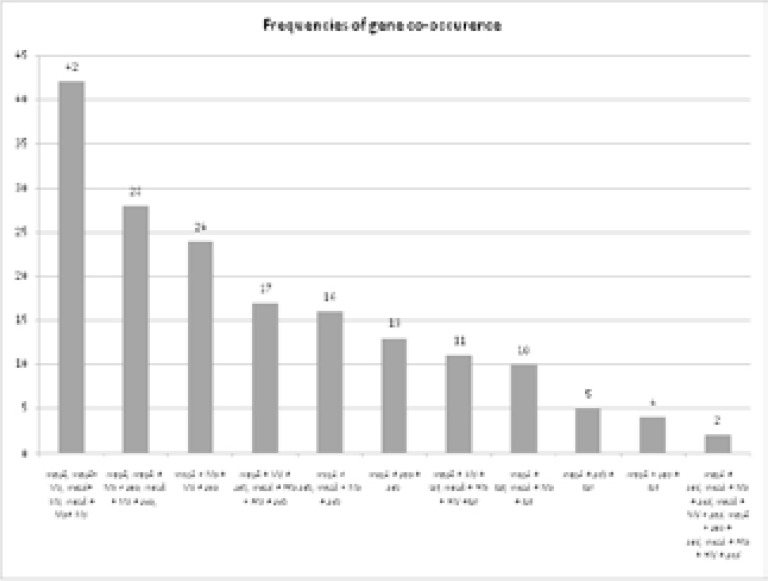
Co-occurrence of *mecA* gene with virulence genes of MRSA isolates.

Figure 4 provides the percentage prevalence of virulence and antibiotic resistance genes in 62 MRSA isolates. The antibiotic resistance gene mecA prevails in Pakistani MRSA strains with high frequency of 69.3%. The virulence markers genes hla and hld were found in all of the strains under study (100% occurrence) whereas sea, seb, sed, tst were found with frequency of 53.22%, 30.64%, 3.22, 24.19%, respectively. The least occurrence was recorded for sed gene that was found in only two out of 62 strains. Figure 5 details the mecA gene co-occurrence with virulence genes of MRSA isolates. In 42 out of 62 samples mecA was present with hla and hld. However, mecA gene co-occurrence was found to be lowest in those isolates which carry sed gene because sed gene itself was found in only two out of 62 strains. None of all these seven genes (mecA, seb, sea, sed, hla, hld, and tst) co-occur in 62 of the isolates under study.

## Discussion

The prolonged, excessive and irrational use of antibiotics has led to the emergence of resistance against once miracle drugs, thus rendering them virtually ineffective against variety of organisms throughout the world. The problem exacerbates with the fact that very few novel classes of antibiotics are in the pharmaceutical development pipeline. First strains of methicillin-resistant *S. aureus* were reported in England in early sixties and since then this particular resistance is on continuous rise in both hospitals and community^2^.

The emergence of resistance against methicillin and other similar antibiotics in *S. aureus* is attributed to the acquisition of mecA gene. Even though the timescales for evolution of resistance and virulence is different yet they share some common characteristics like both of these traits are required for bacterial survival and are transmitted between species or genera through horizontal gene transfer. Dissemination and co-selection of resistant and virulence genes is most probably due to mobile genetic elements. Besides, direct involvement of porins, efflux pumps and cell wall interactions are also common characteristics in resistance and virulence^18^.

Appearance and rapid spread of MRSA in Pakistani hospitals might be attributed to increasing the morbidity and mortality rates; however, this association is under reported. The present study aimed at reporting prevalence of antibiotic resistance (mecA) and virulence (hla, hld, sea, seb, sed, tst) genes, resistance profiling, and investigating relationship between antibiotic resistance and virulence genes among local MRSA isolates. The purpose of this investigation was to understand pathogenic severity of these MRSA isolates as bacterial pathogenicity coupled with higher resistance might hinder the therapeutic management of resultant infections.

The prevalence of MRSA may vary considerably among the regions as reported earlier where MRSA prevalence varied from as low as 2% to as high as 61% in different regions of Pakistan^19^. MRSA prevalence has been reported in varying rates from different cities of Pakistan from 22% in 1999 (Siddique et al.) to 42% in 2002^19^, 42% in 2007^20^, 62% in 2008^21^, 36.1 % in 2016^22^, 52% in 2017^23^ and 70% in 2019^24^.

Detection of mecA is considered as the gold stranded for MRSA confirmation^25^. In the present study, the mecA gene was identified in 69.4% of initially categorized MRSA strains which is comparable to at least two recent reports^24^, ^26^. However, mecA gene was not identified in all the initially categorized MRSA possibly due to the genetic instability of mecA gene during storage at -80°C as reported by^27^ where it was found that 60% of initially categorized MRSA lost their mecA during storage after a period of two years.

MRSA detection through disc diffusion gave reliable results which were confirmed through PCR detection of mecA gene. In the present study, mecA was found to be responsible for making all MRSA strains resistant to various antibiotic groups like penicillin, cephalosporins, tetracycline, macrolides, fluoroquinolones, oxazolidinones, fusidane, sulfonamides and glycopeptides rendering MRSA a multiple drug resistant strain. An absolute resistance observed against Penicillin is comparable to at least one previous report^28^.

In current study, MRSA infection was found more in males (75.9%) than in females (24.1%). A previous study from Peshawar, Pakistan reported 31% MRSA prevalence in females as compared to 69% in males^29^. Contrary to this, similar MRSA prevalence was found in male and female patients from a tertiary care hospital in Rawalpindi^30^. In present study all the age groups were found to be infected with MRSA. Maximum frequency was observed in elderly patients (61–70 years) then younger patients (21–30 years). Lower frequency was found in children less than 10 years of age. So there were two peaks of age groups for *S. aureus* infection. Other groups reported a different pattern of MRSA infections in different age categories for example^26^ reported highest incidences in the age group of 21–40 years. High prevalence at older age may be attributed to generally lower socioeconomic status of Pakistani patients and a generally deficient immune system.

The fact that most MRSA specimens were frequently associated with pus was in conformance with other reports^26^, ^31^. Frequency difference in MRSA virulence genes has been observed in different geographical locations throughout the world. The frequency of sea gene in the present study was found to be 53.2%. There have been varying reports for sea gene profiling across the globe particularly 11.7% from China^32^, 27.4% reported from Iran14 and 78% from India^33^. The prevalence is generally higher in developing countries than the developed world. The frequency of seb gene has also been reported from different countries as 21.4% from China^32^ and 11% reported from Iran^14^. Our finding of 30.6% prevalence of seb gene reflects the same widely varying pattern through different geographical locations.

We reported 3.2% prevalence of sed gene from Pakistani MRSA isolates which is in conformance with reports from other countries that is 1% from China^32^ and 2% from Iran^14^. The frequency of sea and seb in *S. aureus* and their co-existence could be associated with their localization in the enterotoxins gene cluster that is composed of six enterotoxin genes located on the genomic pathogenicity island vSaβ^34^.

Our findings regarding 100% prevalence of hla and hld genes in MRSA isolates are in complete conformance with other reports which found the similar prevalence for hla gene from the USA^35^ and Uganda^36^. Yet other groups have reported higher prevalence for hemolysin genes viz 86.4% (hla) and 53.4% (hld) from China^32^, 93.5% (hla) and 84.2% (hld) from Iran^14^. This conformance reflects that hemolysin genes are comparatively much more frequent in MRSA isolates.

The studies performed on MRSA revealed co-occurrence pattern for antibiotic resistant and virulence genes with considerable fluctuations. In present study the most coexistent genes were hla and hld with mecA gene which is justifiable in the context of highly prevalent hemolysin genes among MRSA isolates from various geographical locations. The lowest coexistence of mecA gene was observed with sed gene. However, none of the isolates possessed all of the seven genes under study.

With increasing antibiotic resistance, the efficiency of antibiotics decreases that leads to situation where most commonly used antibiotics fail to treat routine infections. More complicated and advanced level treatments including cancer therapy, organ surgery, heart transplants, auto-immune diseases, diabetes and joints replacements deped on antibiotics ability to fight the infections. High frequency of virulence and resistant genes in the MRSA isolates is one of a main factor that can not only lead to treatment failure but also spread antibiotic resistance.

## Conclusion

This study showed soaring prevalence of highly virulent and multi drug resistant *S. aureus* in Rawalpindi and its nearby regions in Pakistan especially in aged male patients. All isolates turned out to be resistant to penicillin with alarming resistance against other commonly used antibiotics as well. Co-occurrence of virulence and resistance genes was observed in conformance with reports from the other parts of the world. The situation warrants further research in the form of detailed studies to find out the extent of resistance in the bacteria and levels of antibiotic concentrations in the environment to ascertain the selection pressure posed and to identify the transfer routes to other clinical pathogens. It is also important that use of antibiotics be regulated, restricted and disciplined in order to minimize the mechanisms of resistance development.
